# Interlocus Gene Conversion, Natural Selection, and Paralog Homogenization

**DOI:** 10.1093/molbev/msad198

**Published:** 2023-09-07

**Authors:** Yixuan Yang, Tanchumin Xu, Gavin Conant, Hirohisa Kishino, Jeffrey L Thorne, Xiang Ji

**Affiliations:** Bioinformatics Research Center, North Carolina State University, Raleigh, NC, USA; Bioinformatics Research Center, North Carolina State University, Raleigh, NC, USA; Department of Statistics, North Carolina State University, Raleigh, NC, USA; Bioinformatics Research Center, North Carolina State University, Raleigh, NC, USA; Department of Biological Sciences, North Carolina State University, Raleigh, NC, USA; AI/Data Science Social Implementation Laboratory, Chuo University, Tokyo, Japan; Bioinformatics Research Center, North Carolina State University, Raleigh, NC, USA; Department of Statistics, North Carolina State University, Raleigh, NC, USA; Department of Biological Sciences, North Carolina State University, Raleigh, NC, USA; Department of Mathematics, Tulane University, New Orleans, LA, USA

**Keywords:** interlocus gene conversion, paralog homogenization, teleost genome duplication

## Abstract

Following a duplication, the resulting paralogs tend to diverge. While mutation and natural selection can accelerate this process, they can also slow it. Here, we quantify the paralog homogenization that is caused by point mutations and interlocus gene conversion (IGC). Among 164 duplicated teleost genes, the median percentage of postduplication codon substitutions that arise from IGC rather than point mutation is estimated to be between 7% and 8%. By differentiating between the nonsynonymous codon substitutions that homogenize the protein sequences of paralogs and the nonhomogenizing nonsynonymous substitutions, we estimate the homogenizing nonsynonymous rates to be higher for 163 of the 164 teleost data sets as well as for all 14 data sets of duplicated yeast ribosomal protein-coding genes that we consider. For all 14 yeast data sets, the estimated homogenizing nonsynonymous rates exceed the synonymous rates.

## Introduction

Due to both natural selection and mutation, it has long been appreciated that duplicated regions of the genome may not evolve independently of one another (e.g., Hood et al. 1975; Tartof 1975). One kind of mutation responsible for this lack of independence is known as interlocus gene conversion (IGC) and also as nonallelic gene conversion. An IGC mutation results in a stretch of DNA sequence from one paralog being replaced by the sequence in the corresponding region from another paralog. The evolutionary impact of IGC is difficult to assess because studying it necessitates jointly considering paralogs.

As a result of this difficulty, the possibility of IGC has conventionally been ignored when probabilistic models of sequence change have been employed to characterize molecular evolution or infer phylogenies. One way to avoid consideration of IGC has been to exclusively focus on molecular evolution in single-copy genomic regions. This narrow focus is unfortunate because genomes are often rich in duplicated regions.

While some hypothesized ancient whole-genome duplications are controversial (e.g., Abbasi 2010), it is clear that taxonomically important lineages including angiosperms, teleosts, and yeast experienced them (e.g., Wolfe and Shields 1997; Van de Peer et al. 2009, 2010; Vanneste et al. 2014). For these lineages, genes that are now single copy may have had ancestral paralogs. Therefore, IGC-induced dependence between ancestral paralogs may have shaped the DNA of genes that are single copy in extant genomes.

Rather than ignoring IGC, we have been developing a model-based phylogenetic approach for studying it (Ji et al. 2016). It considers sequence changes that result from both point and IGC mutations. For 14 data sets of ribosomal protein-coding genes that resulted from a historical genome-wide duplication event in the baker’s yeast lineage, the estimated percentage of changes attributable to IGC rather than point mutation ranges from 20% to 38% (Ji et al. 2016). These are percentages of sequence changes due to IGC; they do not reflect sites that were in an IGC tract and that were identical between paralogs both before and after the IGC event. These percentages are especially high when one considers that a homogenizing substitution due to IGC cannot occur unless corresponding codons differ due to a point mutation prior to the IGC event. Therefore, the percentages from IGC cannot exceed 50%. Because yeast ribosomal protein-coding genes are unusually conserved (Kellis et al. 2004) and had been previously recognized for their IGC (Evangelisti and Conant 2010), their high IGC percentages are probably unrepresentative of other duplicated genes.

In contrast, Harpak et al. (2017) examined mammalian intronic regions that were the result of recent duplications. They adopted an inference procedure with similarities to Ji et al. (2016, see also Ji 2017) and concluded that IGC did not have a substantial long-term impact on paralog divergence for their intronic loci. Because so few IGC studies have been done, the overall evolutionary influence of IGC remains uncertain, as do the consequences of ignoring IGC when inferring phylogenies or estimating divergence times.

Motivated by the desire to assess IGC in additional data sets so as to broaden the understanding of how generally relevant IGC is to molecular evolution, we apply and extend our inference procedure to study IGC in 164 protein-coding data sets from teleosts. We conclude that substitutions due to IGC rather than point mutation are responsible for a nonnegligible proportion of all postduplication sequence changes in these data sets. This casts doubt on the usual practice in molecular evolutionary analyses of ignoring IGC following gene duplication.

Beyond establishing the presence of IGC in the teleost data, we also carefully examine the patterns of nonsynonymous change that they have experienced. By analyzing both the teleost data and ribosomal protein-coding genes from yeast, we come to the biologically plausible conclusion that the amino acid that is encoded at a protein position in one paralog is a useful predictor of nonsynonymous rates at the corresponding codon triplet of the other paralog. We conclude by discussing the implications of these nonsynonymous rate patterns, weaknesses of our approach, and promising directions for IGC-related research.

## New Approaches

### The Basic IGC Model

Ji et al. (2016) extended conventional codon-based substitution models to reflect IGC, but a simpler version of the same approach can add IGC to the HKY (Hasegawa et al. 1985) or other four-state nucleotide substitution models. We first review the Ji et al. (2016) approach to facilitate presentation of a generalization that is introduced here. While the IGC model incorporates dependence between corresponding codons of paralogs, it assumes independent evolution among the codons within a paralogous gene. Because IGC mutations homogenize tracts of adjacent sequence positions, actual genome evolution violates this independence assumption. However, the degree of this violation is unknown because it depends on the level of intralocus recombination. When recombination rates are high, the independence assumption may be reasonable because recombination will lessen the dependence in fixation probabilities between sequence sites that comprise an IGC tract. For sufficiently high recombination rates, the IGC inference procedure is effectively a maximum likelihood procedure whereas the approach is more accurately classified as a maximum composite likelihood procedure when recombination rates are low (Ji et al. 2016).

The IGC model describes both codon substitutions that originate with point mutation and those that originate with IGC. Consider corresponding codons of two paralogs, with the first paralog being occupied by a codon triplet denoted by *i* and the second being occupied by a triplet denoted by $i^{\prime }$. For codon substitutions that originate with point mutations, the instantaneous rate of change from triplet state *i* ($i^{\prime }$) to *j* ($j^{\prime }$) in the first (second) paralog will be denoted by $Q_{i,j}$ ($Q_{i^{\prime },j^{\prime }}$). To specify $Q_{i,j}$, any conventional model of nucleotide or codon substitution can be adopted. All analyses conducted for this study have reduced the number of free parameters to be estimated by setting $Q_{i,j}=Q_{i^{\prime },j^{\prime }}$ when $i=i^{\prime }$ and $j=j^{\prime }$.

Because $Q_{i,j}$ represents the rates of codon substitutions that arise from point mutation, $Q_{i,j}=0$ for all triplets *i* and *j* that differ at more than one codon position. As is conventional for codon models, we do not model substitutions that involve stop codons. For *i* and *j* that differ exactly at one position where *j* has nucleotide type *h*, the rates are


(1) $$Q_{i,j}=\left\{ \begin{array}{cc} u\pi_{h} & \text{for a synonymous transversion}\\ u\pi_{h}\kappa & \text{for a synonymous transition}\\ u\pi_{h}\omega & \text{for a nonsynonymous transversion}\\ u\pi_{h}\kappa \omega & \text{for a nonsynonymous transition, } \end{array}\right.$$

with $\pi_{A}+\pi_{C}+\pi_{G}+\pi_{T}=1$ and $0\leq \pi_{h}\leq 1$ for h∈{A,C,G,T}. The *u* in equation (1) is a normalization constant that makes the expected rate per codon of substitutions that arise from point mutation equal to 1. In the notation of the PAML software (Yang 2007), this parameterization of $Q_{i,j}$ is referred to as the F1×4MG+κ+ω model.

For a genetic code with 61 possible codon states, joint consideration of the two paralogs requires specification of the instantaneous rate of change $Q_{\left(i,i^{\prime }),(j,j^{\prime }\right)}$ from the $61^{2}$ possible combinations of *i* and $i^{\prime }$ to each of the $61^{2}$ possible combinations of *j* and $j^{\prime }$. Our joint substitution model with IGC has:


(2) $$Q_{\left(i,i^{\prime }),(j,j^{\prime }\right)}=\left\{ \begin{array}{cc} 0 & i\neq j,\;i^{\prime }\neq j^{\prime }\\ Q_{i,j} & i\neq j,\;i^{\prime }=j^{\prime },\;j\neq j^{\prime }\\ Q_{i^{\prime },j^{\prime }} & i=j,\;i^{\prime }\neq j^{\prime },\;j\neq j^{\prime }\\ Q_{i,j}+\nu & i\neq j,\;i^{\prime }=j^{\prime },\;j=j^{\prime }\\ Q_{i^{\prime },j^{\prime }}+\nu & i=j,\;i^{\prime }\neq j^{\prime },\;j=j^{\prime }, \end{array}\right.$$

where ν=τ if the change is synonymous and where ν=ωτ if the change is nonsynonymous. The parameter *τ* controls the amount of IGC. Our practice is to normalize rates to make the expected rate per codon equal to one for substitutions that arise from point mutation. This normalization is performed for the case where IGC is absent (i.e., τ=0) and we do not renormalize to make the normalization dependent on the value of *τ*. This way of normalizing rates leads to an intuitive interpretation of the value of *τ*. For sites that differ between paralogs, τ=1 implies that the rate of homogenization due to IGC is approximately equal to the rate of substitutions that originate with point mutation. The approximation is not perfect because IGC events can simultaneously change multiple nucleotides when a codon differs at more than one position in the two paralogs.

### The $\omega_{H}/\omega_{N}$ IGC Model

For the Ji et al. (2016) model, excess homogenization of amino acid types at corresponding positions in two paralogs is explained by IGC. Here, excess homogenization refers to more homogenization than would be expected for independently evolving paralogs. An alternative—but not mutually exclusive—explanation for excess homogenization could be homogenizing nonsynonymous point mutations having higher fixation probabilities than nonhomogenizing nonsynonymous point mutations. This alternative explanation might be biologically plausible when the fitness effects of amino acid types are positively correlated at corresponding sites in paralog proteins. These competing explanations can be evaluated by fitting models with or without IGC and with or without an excess of fixation for homogenizing nonsynonymous point mutations.

We therefore generalize the codon substitution rates of equation (1) by having the amino acid encoded by a codon in one paralog influence the nonsynonymous rates of the corresponding codon triplet in the other paralog. Specifically, we replace the *ω* parameter by $\omega_{H}$ when the nonsynonymous change homogenizes amino acids that are encoded by corresponding codons in the two paralogs. Similarly, we replace *ω* by $\omega_{N}$ when the nonsynonymous change is nonhomogenizing.

As with the Ji et al. (2016) model, this model has synonymous changes due to IGC occur at rate *τ*. Because all IGC events homogenize paralogs, this model has rate $\omega_{H}\tau$ for all nonsynonymous IGC change. The rates of codon substitution that originate with a point mutation become


(3) $$Q_{i,j}=\left\{ \begin{array}{cc} u\pi_{h} & \text{for a synonymous transversion}\\ u\pi_{h}\kappa & \text{for a synonymous transition}\\ u\pi_{h}\omega_{H} & \text{for a nonsynonymous homogenizing transversion}\\ u\pi_{h}\omega_{N} & \text{for a nonsynonymous nonhomogenizing transversion}\\ u\pi_{h}\kappa \omega_{H} & \text{for a nonsynonymous homogenizing transition}\\ u\pi_{h}\kappa \omega_{N} & \text{for a nonsynonymous nonhomogenizing transition.} \end{array}\right.$$

In equation (3), note that $\omega_{H}$ is used for nonsynonymous changes that cause the nucleotide triplets in corresponding codon positions of the two paralogs to encode the same amino acid even when the triplets differ at the nucleotide level. The value of *u* is again set so that the expected rate of substitution per codon equals one in the absence of IGC (i.e., when all substitutions are attributable to point mutation). However, the rate of change for this model at a codon position in one paralog depends on the encoded amino acid at the corresponding position in the other paralog. For the standard genetic code, normalizing to find the value of *u* that makes the expected rate equal to one involves considering a rate matrix with $61^{2}$ rows and columns.

When considering codon substitutions that originate with a point mutation, we denote the rates of equation (3) as the $\omega_{H}/\omega_{N}$ model. The special case of $\omega_{H}=\omega_{N}$ that yields the rates of equation (1) is denoted as the *ω* model. Because these two treatments of point mutation can each be adopted with or without IGC, the four model possibilities are termed: $\omega_{H}/\omega_{N}+\mathrm{IGC}$, $\omega_{H}/\omega_{N}-\mathrm{IGC}$, ω+IGC, and ω−IGC.

## Results

### Duplicated Teleost Genes

**Fig. 1.**
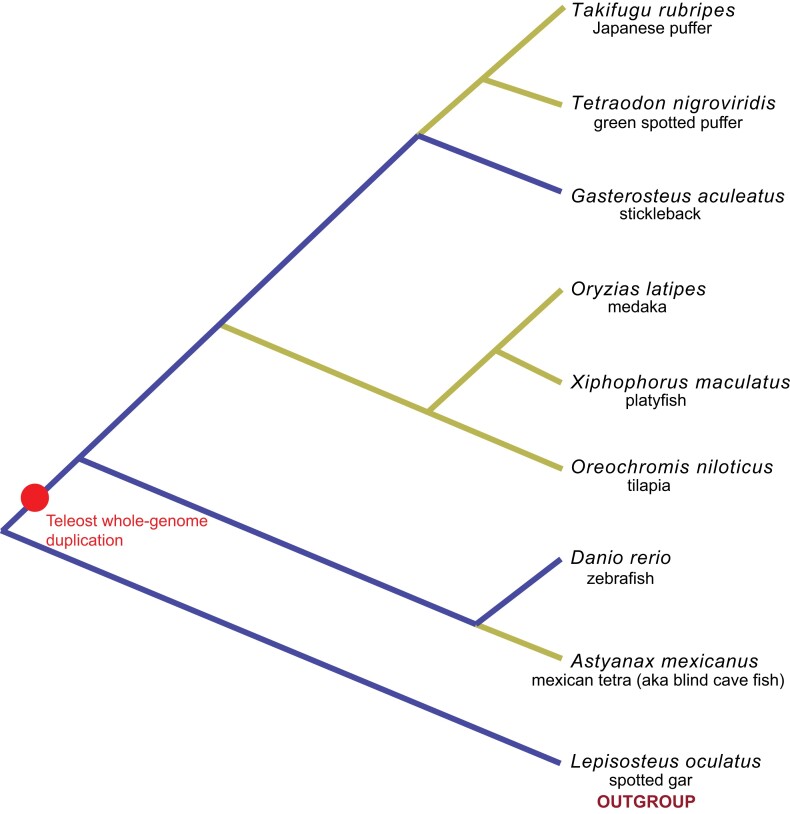
Species tree of the teleosts in this study. For the subtree shown in blue (i.e., subtree that includes *Lepisosteus oculatus, Danio repio*, and *Gasterosteus aculeatus*), all 164 data sets have representative sequences (i.e., two paralogs each from zebrafish and stickleback plus one sequence from the outgroup gar). For the postduplication taxa that are connected to the subtree via branches colored yellow (i.e., *Astyanax mexicanus, Oreochromis niloticus, Xiphophorus maculatus, Oryzias latipes, Tetraodon nigroviridis, *and* Takifugu rubripes*), some data sets include two paralogs and others do not include any.

Most teleost species are descended from a whole-genome duplication that occurred between 300 and 400 million years ago (e.g., Christoffels et al. 2004; Vandepoele et al. 2004; Crow et al. 2006). Conant (2020) inferred syntenic relationships between the genomes of eight postduplication teleosts and an outgroup. The species tree relating the taxa from that study is depicted in figure 1.

Here, we characterize IGC that has occurred subsequent to this teleost genome duplication (TGD) by relying upon the orthology/paralogy inferences of Conant (2020) to collect 164 paralogy groups. All 164 data sets have a sequence from the outgroup taxon. For 37 of the 164 data sets, all 8 post-TGD taxa have both paralog sequences. The remaining 127 data sets do not include both paralogs from all 8 post-TGD taxa, presumably because of deletion of one or both paralogs subsequent to the TGD event. These 127 data sets were selected based on 2 paralogs being available from both the stickleback and zebrafish genomes. For each of the other 6 post-TGD taxa considered by Conant (2020), sequences from that species are included in a data set only when 2 paralog sequences are available. Further details and justification regarding our procedure for collecting the data sets can be found in Materials and Methods section. For the 164 teleost data sets, alignment lengths and some estimated parameter values for the $\omega_{H}/\omega_{N}+\mathrm{IGC}$ model are listed in supplementary table S1, Supplementary Material online: Teleosts.

### Duplicated Yeast Genes

Ji et al. (2016) quantified the evolutionary impact of IGC in 14 groups of yeast ribosomal protein-coding genes that resulted from an ancient genome duplication. These data sets each consist of a single sequence from an outgroup taxon as well as two paralogs from each of 6 yeast species that are descended from the ancient whole-genome duplication event. Here, we analyze the 14 data sets from Ji et al. (2016) with the $\omega_{H}/\omega_{N}$ model. For the 14 yeast data sets, alignment lengths and some estimated parameter values for the $\omega_{H}/\omega_{N}+\mathrm{IGC}$ model are listed in supplementary table S2, Supplementary Material online: Yeast.

### Model Comparison

We can assess four different models with the most flexible being the $\omega_{H}/\omega_{N}+\mathrm{IGC}$ model and the simplest being the ω−IGC model. The $\omega_{H}/\omega_{N}-\mathrm{IGC}$ and ω+IGC models represent intermediates between these extremes. Likelihood ratio tests that use twice the difference between maximum log-likelihood values as a test statistic are available for comparing different pairs of these models.

To assess a null hypothesis that homogenizing and nonhomogenizing nonsynonymous rates are equal (i.e., $\omega_{H}=\omega_{N}$) versus an alternative without the equality constraint, the null distribution of the likelihood ratio test statistic is approximately (i.e., asymptotically) a $\chi_{1}^{2}$ random variable when codon positions are assumed to independently evolve. For the case of testing the ω+IGC model as the null hypothesis and the $\omega_{H}/\omega_{N}+\mathrm{IGC}$ model as the alternative, the $\chi_{1}^{2}$ test statistic would not be appropriate when recombination is low enough to mean that our inferences under the null hypothesis need to be considered as maximum composite likelihood estimates rather than maximum likelihood estimates. Specifically, the test statistic is based on the assumption that IGC substitutions occur independently of one another. This would not be a good assumption if individual IGC tracts are likely to homogenize multiple codons that differed in sequence prior to the IGC event and if recombination rates are low enough so that the different resulting homogenized codons are likely to be jointly fixed. Here, we assume that recombination rates are sufficiently high.

To assess the null hypothesis that τ=0 versus the alternative that *τ* is free to vary, the fact that τ=0 is at the boundary of the parameter space should be considered. In this case, the null distribution of the test statistic is approximately an equiprobable mixture of $\chi_{0}^{2}$ and $\chi_{1}^{2}$ distributions where the $\chi_{0}^{2}$ distribution has value 0 with probability 1 (Self and Liang 1987; Goldman and Whelan 2000; Ota et al. 2000). To compare the null hypothesis that τ=0 and also $\omega_{H}=\omega_{N}$ versus an alternative that has neither of these constraints, the appropriate test statistic is an equiprobable mixture of $\chi_{1}^{2}$ and $\chi_{2}^{2}$ random variables.

One way to decompose the test statistic for comparing the ω−IGC and $\omega_{H}/\omega_{N}+\mathrm{IGC}$ models is as the sum of the test statistic for comparing the ω−IGC and ω+IGC models and the test statistic for comparing the ω+IGC and $\omega_{H}/\omega_{N}+\mathrm{IGC}$ models. This decomposition represents a path from the simplest (ω−IGC) model to the most flexible model ($\omega_{H}/\omega_{N}+\mathrm{IGC}$) by first adding IGC to the simplest model to get the intermediate ω+IGC model and then distinguishing between homogenizing and nonhomogenizing nonsynonymous change to obtain the $\omega_{H}/\omega_{N}+\mathrm{IGC}$ model. An alternative decomposition for comparing the ω−IGC and $\omega_{H}/\omega_{N}+\mathrm{IGC}$ models is as the sum of the test statistic for comparing the ω−IGC and $\omega_{H}/\omega_{N}-\mathrm{IGC}$ models and the test statistic for comparing the $\omega_{H}/\omega_{N}-\mathrm{IGC}$ and $\omega_{H}/\omega_{N}+\mathrm{IGC}$ models. This alternative decomposition represents a path from the simplest to the most flexible model by first distinguishing between the two sorts of nonsynonymous change to yield the intermediate $\omega_{H}/\omega_{N}-\mathrm{IGC}$ model and then adding IGC to obtain the $\omega_{H}/\omega_{N}+\mathrm{IGC}$ model.

**Fig. 2.**
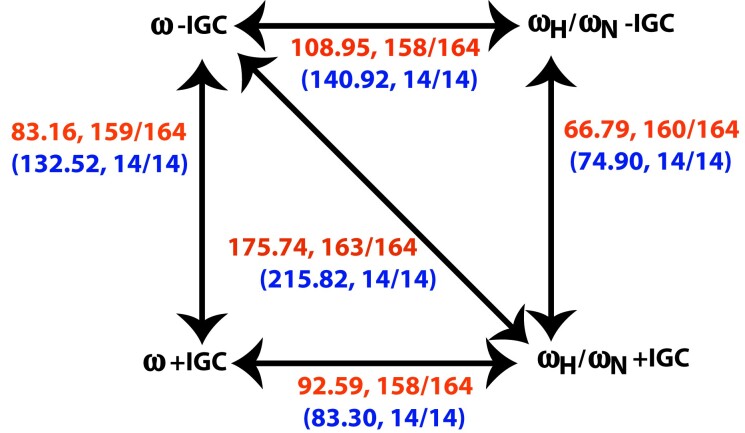
Bidirectional arrows indicate model comparisons that were performed. For each comparison, the top line summarizes results from the 164 teleost data sets and the parenthesized bottom line has results from the 14 yeast ribosomal protein-coding data sets. Each line contains the sample mean among the data sets of the test statistic (i.e., twice the log-likelihood differences between models) followed by the proportion of the data sets for which the null hypothesis was rejected at a significance level of 0.05.

Figure 2 summarizes the results of hypothesis tests between our models for the teleost and yeast data. The mean log-likelihood improvement among data sets is substantial for each step in each of the 2 two-step paths for decomposing the comparison of the ω−IGC and $\omega_{H}/\omega_{N}+\mathrm{IGC}$ models via an intermediate model (fig. 2). The substantial improvements arise both on each step of the two-step path where IGC is first added to the simplest model and on each step of the two-step path where the differentiation between nonsynonymous substitution types is first added. These results suggest that IGC and differentiation of nonsynonymous substitution types are not completely redundant and that both features are important. However, figure 2 also demonstrates that there is some interaction between the features. For example, the mean log-likelihood improvement from adding IGC is higher on the two-step path where IGC is added first than on the two-step path where IGC is added second.

**Fig. 3.**
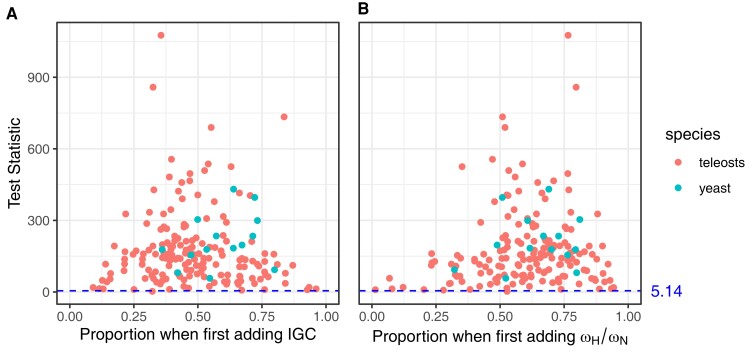
Likelihood ratio test statistics for the teleost and yeast data sets when comparing the ω−IGC model to intermediate models versus when comparing the ω−IGC model to the $\omega_{H}/\omega_{N}+\mathrm{IGC}$ model. The *x*-axes represent ratios of likelihood ratio test statistics (i.e., twice the difference between the maximum log-likelihood of the alternative and null hypotheses). The numerator of these ratios is the test statistic when the null hypothesis is the ω−IGC model and the alternative is an intermediate model with one additional free parameter. The denominator of these ratios is the test statistic when the null hypothesis is the ω−IGC model and the alternative is the $\omega_{H}/\omega_{N}+\mathrm{IGC}$ model that has two additional free parameters. The *y*-axes represent the test statistic in the denominator of the ratio (i.e., the null hypothesis is the ω−IGC model and the alternative is the $\omega_{H}/\omega_{N}+\mathrm{IGC}$ model). For the test statistics displayed on the *y*-axes, horizontal lines indicate the critical value of y=5.14 at the 0.05 significance level. (*A*) The intermediate model is ω+IGC. (*B*) The intermediate model is $\omega_{H}/\omega_{N}-\mathrm{IGC}$.

While figure 2 summarizes the mean behavior among data sets of test statistics, figure 3 displays the behavior for individual data sets. When IGC is added to the ω−IGC model to produce the intermediate ω+IGC model, the improvement in log-likelihood tends to be big but tends to represent only a moderate proportion of the total improvement in log-likelihood between the ω−IGC and $\omega_{H}/\omega_{N}+\mathrm{IGC}$ models (fig. 3*A*). Similarly, when differentiating between the two types of nonsynonymous change is added to the ω−IGC model to produce the intermediate $\omega_{H}/\omega_{N}-\mathrm{IGC}$ model, the improvement in log-likelihood tends to be big but again tends to represent only a moderate proportion of the total improvement in log-likelihood between the ω−IGC and $\omega_{H}/\omega_{N}+\mathrm{IGC}$ models (fig. 3*B*).

### IGC Proportions

For branches that are subsequent to the first postduplication speciation event, sequence differences between paralogs at the beginning of the branches can be employed to detect IGC that occurs later on the branches. In contrast, paralog sequences are identical immediately after a duplication. Codon substitutions that occur after a duplication but that are homogenized by IGC before the first postduplication speciation are not easily differentiated from codon substitutions that occur on the branch prior to the duplication. As explained in Ji et al. (2016), our phylogenetic approach therefore has difficulty in extracting IGC information from branches separating a duplication event from the first postduplication speciation. It also has difficulty estimating the expected number of codon substitutions that occurred on these branches. As demonstrated by simulation, evolutionary inferences regarding these initial postduplication branches tend to be overly sensitive to model assumptions. When we estimate the proportion of codon substitutions that arise via IGC rather than point mutation, we therefore only consider the branches on the species tree that occur subsequent to the first postduplication speciation.

**Fig. 4.**
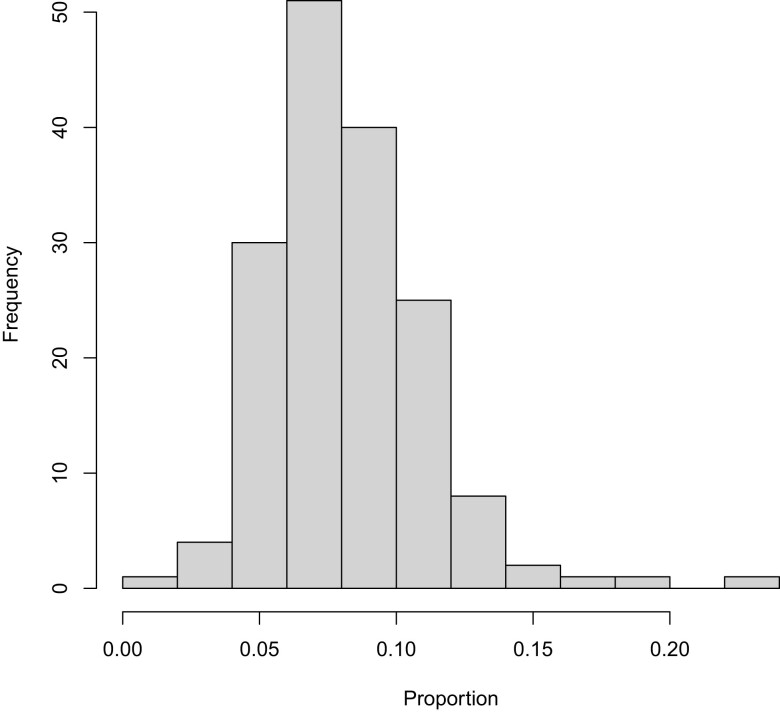
A histogram of the estimated proportions of codon substitutions that are due to IGC for the 164 teleost data sets when the ω+IGC model is assumed.

As shown in Ji et al. (2016), these proportions can be inferred via the “matrix of exponentials” technique of Tataru and Hobolth (2011). Using the Tataru–Hobolth technique on each of our 164 teleost data sets, the estimated IGC proportions when assuming the ω+IGC model range among data sets from slightly less than 0.02 to slightly more than 0.22 with a median value of about 0.08. Figure 4 depicts the distribution of these estimated proportions. The estimated proportion of codon substitutions due to IGC rather than point mutation is relatively insensitive to whether the ω+IGC model or the $\omega_{H}/\omega_{N}+\mathrm{IGC}$ model is assumed. For the teleost genes under the $\omega_{H}/\omega_{N}+\mathrm{IGC}$ model, the estimated proportions range from slightly more than 0.01 to slightly less than 0.22 with a median value between 0.07 and 0.08, a reduction of about 0.005 in the median relative to the ω+IGC model.

### Estimates of $\omega_{H}$ and $\omega_{N}$

**Fig. 5.**
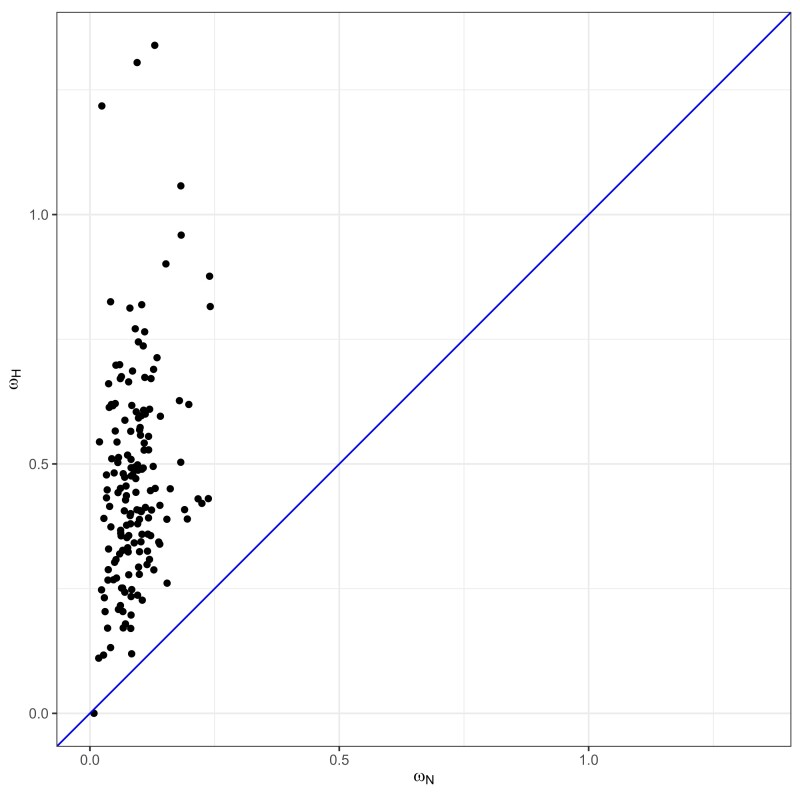
A plot of the estimated $\omega_{N}$ (*x*-axis) and $\omega_{H}$ (*y*-axis) values for the 164 teleost data sets when assuming the $\omega_{H}/\omega_{N}+\mathrm{IGC}$ model. The diagonal line represents y=x.

Figure 5 plots estimates of $\omega_{N}$ versus $\omega_{H}$ from the 164 teleost data sets when assuming the $\omega_{H}/\omega_{N}+\mathrm{IGC}$ model. The $\omega_{N}$ estimates were all between approximately 0.01 and 0.24 with a median of about 0.08. For 159 of the 164 teleost data sets, the $\omega_{H}$ and $\omega_{N}$ estimates had $0<\omega_{N}<\omega_{H}<1$. One data set violated this pattern in that the estimated $\omega_{N}$ was small and the estimated $\omega_{H}$ was even closer to 0. The remaining 4 data sets violated the pattern in that each yielded an $\omega_{H}$ estimate that was above 1.0, with all 4 estimates being between 1.0 and 1.4.

In contrast, the 14 yeast data sets all have estimates with the $\omega_{H}/\omega_{N}+\mathrm{IGC}$ model that satisfy $0<\omega_{N}<1<\omega_{H}$. The yeast $\omega_{N}$ estimates range from approximately 0.02 to 0.17 with a median of 0.08. The smallest of the yeast $\omega_{H}$ estimates is 1.36, a value that slightly exceeds the largest of the 164 $\omega_{H}$ estimates from teleosts. The median $\omega_{H}$ estimate from yeast is 5.70. Two of the 14 $\omega_{H}$ estimates are ∞. For these two data sets, the encoded amino acids of the two paralogs are identical within species but different between species. However, paralogs for these two data sets do have some within-species synonymous variation.

The $\omega_{N}$ estimates are not greatly affected by whether the $\omega_{H}/\omega_{N}+\mathrm{IGC}$ or the $\omega_{H}/\omega_{N}-\mathrm{IGC}$ model is assumed, with the yeast data sets showing more sensitivity than the teleost data sets. For the teleosts, subtracting the $\omega_{N}$ estimate of the $\omega_{H}/\omega_{N}-\mathrm{IGC}$ model from that of the $\omega_{H}/\omega_{N}+\mathrm{IGC}$ model yields differences that range from approximately −0.007 to 0.014 with a median that is very close to 0. For the yeast data sets, 13 of the 14 $\omega_{N}$ differences are positive but all are still relatively close to 0. Specifically, the $\omega_{N}$ differences for yeast range from about −0.015 to 0.030 with a median of 0.008. Similarly, when the $\omega_{N}$ estimate from the $\omega_{H}/\omega_{N}-\mathrm{IGC}$ model is divided by the $\omega_{N}$ estimate from the $\omega_{H}/\omega_{N}+\mathrm{IGC}$ model, the median ratio is approximately 0.997 among the teleost data sets and 0.864 among the yeast data sets.

**Fig. 6.**
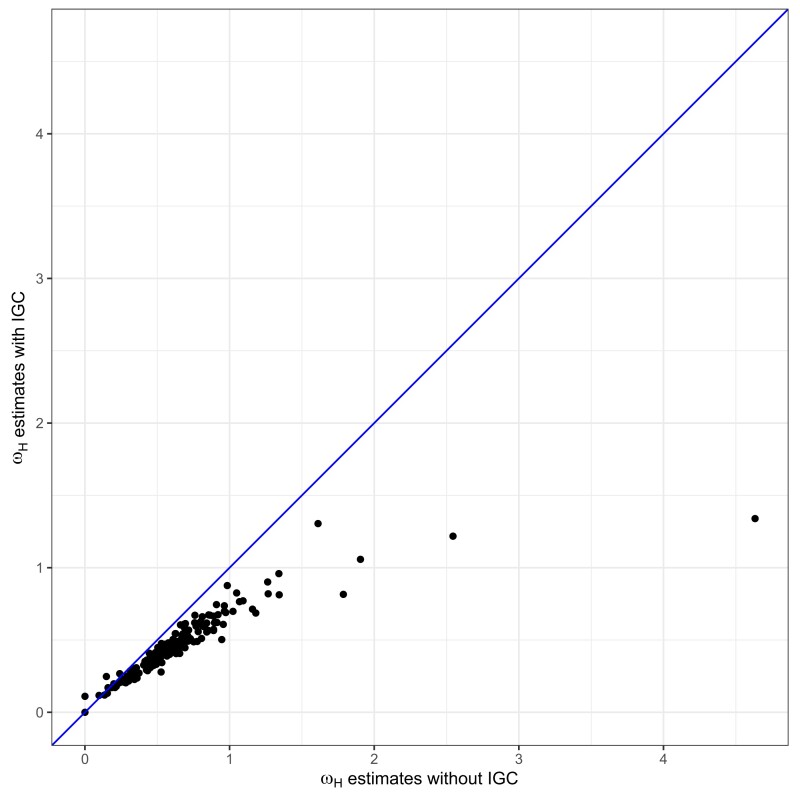
The estimates of $\omega_{H}$ from the 164 teleost data sets when assuming the $\omega_{H}/\omega_{N}-\mathrm{IGC}$ model (*x*-axis) versus the estimates of $\omega_{H}$ when assuming the $\omega_{H}/\omega_{N}+\mathrm{IGC}$ model (*y*-axis).

The $\omega_{H}$ estimates are quite sensitive to whether the $\omega_{H}/\omega_{N}+\mathrm{IGC}$ or the $\omega_{H}/\omega_{N}-\mathrm{IGC}$ model is assumed. For the teleost data sets, figure 6 plots the $\omega_{H}$ estimates from the $\omega_{H}/\omega_{N}-\mathrm{IGC}$ model versus the estimates from the $\omega_{H}/\omega_{N}+\mathrm{IGC}$ model. In 158 of the 164 cases, the $\omega_{H}$ estimates are larger from the $\omega_{H}/\omega_{N}-\mathrm{IGC}$ model. Figure 6 shows that the disparity in $\omega_{H}$ estimates grows as the estimated values of $\omega_{H}$ from the $\omega_{H}/\omega_{N}-\mathrm{IGC}$ model get larger. For the yeast data sets, the $\omega_{H}$ estimates from the $\omega_{H}/\omega_{N}-\mathrm{IGC}$ model are especially large and are presumably too large to be stable estimates. For the $\omega_{H}/\omega_{N}-\mathrm{IGC}$ case, our results had all 14 yeast $\omega_{H}$ values exceeding 5 and had 12 of the 14 estimates exceeding 20.

### Nonsynonymous IGC Rates

The $\omega_{H}/\omega_{N}+\mathrm{IGC}$ model has the nonsynonymous IGC rate equal to $\omega_{H}\tau$, whereas the ω+IGC model has it being ωτ. For these two models, table 1 summarizes estimates of parameters most relevant to nonsynonymous rates. Table 1 shows that the estimated nonsynonymous IGC rate is sensitive to which of these two models is being assumed. For the $\omega_{H}/\omega_{N}+\mathrm{IGC}$ model, the median $\hat{\omega_{H}}\hat{\tau }$ is approximately 0.07 for the teleost data sets, while the median $\hat{\omega }\hat{\tau }$ is approximately 0.02 for the ω+IGC model. This disparity is mainly attributable to the $\omega_{H}$ estimates from $\omega_{H}/\omega_{N}+\mathrm{IGC}$ being higher than the *ω* estimates from ω+IGC. For teleosts, the median $\hat{\omega_{H}}$ is 0.44 for $\omega_{H}/\omega_{N}+\mathrm{IGC}$ and the median $\hat{\omega }$ is 0.10 for ω+IGC. In contrast, the τ estimates between the two models are more similar with the median *τ* estimate for teleosts being about 0.18 for $\omega_{H}/\omega_{N}+\mathrm{IGC}$ and about 0.22 for ω+IGC. In addition, the teleost estimates of $\omega_{N}$ for $\omega_{H}/\omega_{N}+\mathrm{IGC}$ tend to be similar to estimates of ω for ω+IGC. Among teleost data sets, the median ratio of $\hat{\omega_{N}}$ from $\omega_{H}/\omega_{N}+\mathrm{IGC}$ to $\hat{\omega }$ from ω+IGC is about 0.90.

**Table 1.** Estimates of Parameters Relevant to Nonsynonymous IGC Rates from the 14 Yeast and the 164 Teleost Data Sets.

	ω+IGC			$\omega_{H}/\omega_{N}+IGC$			
	$\hat{\omega }$	$\hat{\tau }$	$\hat{\omega }\hat{\tau }$	$\hat{\omega_{N}}$	$\hat{\omega_{H}}$	$\hat{\tau }$	$\hat{\omega_{H}}\hat{\tau }$
Yeast	0.09	2.99	0.26	0.08	5.70	2.30	8.75
	(0.07,0.15)	(1.68,3.79)	(0.15,0.46)	(0.08,0.12)	(2.34,8.02)	(1.30,3.12)	(6.72,21.54)
Teleost	0.10	0.22	0.02	0.08	0.44	0.18	0.07
	(0.07,0.12)	(0.17,0.28)	(0.01,0.03)	(0.06,0.11)	(0.33,0.58)	(0.12,0.24)	(0.05,0.11)

Note.—Each entry is a median among estimates for the data sets. Parenthesized values below the medians represent the lower and upper quartiles.

For the 14 yeast data sets, estimates of τ and $\omega_{H}$ tend to be substantially higher than is the tendency for the teleost data sets. However, the median estimates of $\omega_{N}$ are quite similar between the yeast and teleosts. The yeast inferences also resemble those from the teleosts in that the *ω* estimates from the ω+IGC model are close to the $\omega_{N}$ estimates from the $\omega_{H}/\omega_{N}+\mathrm{IGC}$ model.

## Discussion

### Estimates of IGC and Nonsynonymous Rates

There is strong evidence for IGC in nearly all teleost data sets. This evidence is compelling whether or not homogenizing and nonhomogenizing nonsynonymous changes are separately treated. While it is unclear whether teleost genes experienced enough IGC to have an important influence on phylogeny inference or divergence time estimation, we view the quantification of IGC as being fundamentally necessary for the study of molecular evolution.

The estimated proportion of codon substitutions from IGC is relatively robust to how we model nonsynonymous rates. We believe this robustness is explained by much of the information about IGC coming from homogenizing synonymous substitutions. However, the estimates of $\omega_{H}$ are sensitive to whether or not IGC is allowed. Presumably, this sensitivity arises because IGC is responsible for some of the homogenizing nonsynonymous changes and those can only be explained by a large value of $\omega_{H}$ when IGC is ignored.

Except for 5 of the 164 teleost data sets, our parameter estimates have $0<\omega_{N}<\omega_{H}<1$. For yeast ribosomal protein-coding genes, all 14 data sets yielded $0<\omega_{N}<1<\omega_{H}$. For all cases, the relative values of $\omega_{H}$ and $\omega_{N}$ estimates should be cautiously interpreted as should whether or not these estimates exceed 1. However, the fact that $\omega_{N}$ and $\omega_{H}$ differ establishes that the evolution of one paralog can illuminate the evolution of another. Multiple explanations for inferring $\omega_{H}>\omega_{N}$ are possible.

Most obviously, there may be variation of relative fitnesses of amino acid types at a protein position. If the relative fitnesses of amino acid types are positively correlated at corresponding positions in two paralogs, homogenizing nonsynonymous mutations could be more likely to yield selective advantages than nonhomogenizing mutations and this could result in $\omega_{H}>\omega_{N}$. Importantly, the existence of positive correlations of relative fitnesses of amino acid types among paralogs could be consistent with either $0<\omega_{N}<\omega_{H}<1$ or $0<\omega_{N}<1<\omega_{H}$. All else equal, $0<\omega_{N}<\omega_{H}<1$ might be expected when the positive correlation is relatively weak and $0<\omega_{N}<1<\omega_{H}$ might occur when the positive correlation is strong. A positive correlation is biologically plausible because corresponding protein positions of paralogs are likely to be under similar selective constraints. Perhaps, positive correlations would diminish over evolutionary time as paralog functions diverge.

A distinct issue is to understand why the yeast ribosomal protein-coding data sets suggest $0<\omega_{N}<1<\omega_{H}$, whereas the teleost pattern tends to be $0<\omega_{N}<\omega_{H}<1$. One might expect that ribosomal protein-coding genes are highly expressed and therefore they experience strong selection that leads to $\omega_{H}>1$. Although the median $\omega_{N}$ estimate among teleost data sets and the median $\omega_{N}$ estimate among yeast data sets are not very different, this similarity is complicated by the fact that codon usage bias is strong in yeast ribosomal protein-coding genes (Moriyama and Powell 1998) and this means that the relative rates of synonymous and nonsynonymous change are both affected by substantial natural selection.

Evangelisti and Conant (2010) noted that the expression patterns of paralogs of ribosomal proteins can substantially differ even when the encoded proteins have highly similar amino acid sequences. They attributed this type of subfunctionalization to the ribosome being “a tightly integrated functional module” with the paralogs needing to be “able to substitute for each other under the different expression conditions.” This need for paralog proteins to substitute for one another in the ribosome may be at least a partial explanation for why $\omega_{H}>1$ for all 14 yeast data sets.

An attractive future research direction might be to subdivide the $\omega_{N}$ nonhomogenizing nonsynonymous parameter into two cases, with one being for situations where the two corresponding encoded amino acids differ both before and after a change and with another being for situations where the encoded amino acids are identical before the change but differ after it. Likewise, it seems to be worthwhile to extend promising mutation-selection balance models that allow variation of preferred amino acid types among sites (e.g., see Halpern and Bruno 1998; Tamuri et al. 2009; Rodrigue et al. 2010) to include correlations between paralogs in preferred amino acid types among corresponding positions. One can also envision developing model-based strategies that distinguish between homogenizing and nonhomogenizing nonsynonymous changes when attempting to characterize paralog evolution with respect to nonfunctionalization, neofunctionalization, subfunctionalization, or functional stasis. The basic idea would be that existing and widely used strategies for parameterizing nonsynonymous rates could be supplemented by explicitly including homogenizing nonsynonymous substitution rates that vary among sites and/or lineages.

### Tetrasomic Inheritance Versus IGC

An evolutionary genomic study by Parey et al. (2022) persuasively suggests that the whole-genome duplication in teleosts was an autopolyploid event that was followed by a period of tetrasomic inheritance and then rediploidization to yield the paralogs that are found in extant genomes. The Parey et al. (2022) work further indicates that this rediploidization occurred at different times in different portions of ancestral teleost genomes. Importantly, the Parey et al. (2022) evidence for tetrasomic inheritance does not extend as late as the first postduplication speciation event relating the taxa that we studied. Because our phylogenetic approach for quantifying IGC is only able to infer IGC that occurs subsequent to the first postduplication speciation among the taxa in a sample (Ji et al. 2016), the tetrasomic inheritance detected by Parey et al. (2022) does not explain our results. A hypothesis that has tetrasomic inheritance extended beyond the periods detected by Parey et al. (2022) and beyond the first postduplication speciation among our taxa would face the challenge of explaining why there seems to be a nonnegligible IGC impact for nearly all of the 164 teleost data sets, even though these data sets represent diverse genomic regions.

### Future Directions for Studying IGC

Techniques for studying the intersection of IGC and evolution remain primitive. The approach used here can be classified as a composite likelihood procedure unless recombination rates are sufficiently high. Ideally, the approach would not ignore the fact that IGC mutations affect stretches of consecutive sequence positions. Although the approach yielded reasonable parameter estimates even when data were simulated by having IGC events affect sequence tracts (Ji et al. 2016), it can be challenging to measure the uncertainty of parameters estimated with composite likelihood approaches (e.g., Varin et al. 2011). Better handling of IGC tracts is needed, especially because length distributions of IGC tracts may vary across genomes and among species (e.g., see Mansai et al. 2011).

Furthermore, the IGC inference approach is computationally challenging because the joint consideration of paralogs greatly increases the state space associated with matrices of evolutionary rates. This is the reason why the analyses performed here have been restricted to two paralogs per genome. Monte Carlo data augmentation strategies are one possibility for studying molecular evolution when more than two paralogs per genome are considered.

Another attractive research direction would account for the relationship between IGC rates and paralog divergence. Gene conversion rates decrease as paralog divergence increases (e.g., Chen et al. 2007), but a refined characterization of this relationship is unavailable and should be pursued. Finally, it is unclear whether the evolutionary impacts of IGC have taxonomic-specific patterns and whether the impact is different for segmental and whole-genome duplications.

## Materials and Methods

### Teleost Data Set Collection

Conant (2020) employed the POInT software (Conant and Wolfe 2008; Emery et al. 2018) to estimate orthology/paralogy relationships among protein-coding genes in the genomes of eight representative species that are descended from the TGD. Conant (2020) linked these paralog sets to genes from the spotted gar (*Lepisosteus oculatus*), a representative of an outgroup lineage that diverged from other teleosts prior to the whole-genome duplication. The POInT software assigns confidence scores to its best estimate of orthology/paralogy relationships at homologous genomic loci that are descended from a whole-genome duplication. For our teleost analyses, we considered only protein-coding loci with a confidence score that equaled or exceeded 95%.

The implementation of our IGC model does not account for the possibility of paralog deletion following duplication. Proper handling would include the possible influence via IGC of deleted paralogs on surviving ones. For the data sets that we analyzed, we therefore only included the subset of postduplication taxa for which both paralogs were present.

We further restricted our investigations to cases with a single identifiable homolog in the outgroup gar genome and with two paralogs in both the postduplication zebrafish and the postduplication three-spined stickleback. This requirement was instituted because zebrafish and stickleback are representatives of the earliest postduplication speciation event on the phylogeny relating our taxa (see fig. 1). Because our phylogenetic approach relies on evolution subsequent to the earliest postduplication speciation event to characterize IGC, we did not want to include data sets where the earliest postduplication speciation was later than the zebrafish–stickleback split.

Each protein-coding DNA data set was further processed by translating the sequences and aligning the resulting amino acid sequences with Version 13.45.0.4846264 of the T-Coffee software (Notredame et al. 2000). Amino acids in the aligned data were then replaced with the corresponding codon triplets. All alignment columns with at least one gapped position were removed prior to subsequent analysis.

We eliminated potentially problematic data sets by establishing two additional criteria. Both criteria involved maximum likelihood estimation with the ω−IGC model. The first criterion eliminated data sets with at least one overly long estimated branch length. Our motivation was that long branch length estimates could stem from sequencing or assembly error, alignment mistakes, and/or paralogy/orthology misidentification. Specifically, a data set was removed from further consideration if any estimated branch lengths exceeded 1.5 codon substitutions per codon.

The second criterion only involved 5 sequences from each of the 164 teleost data sets. Because all data sets include two paralogs from both stickleback and zebrafish as well as one sequence from gar, we compared the maximum log-likelihood value for these five sequences being related via the paralogy/ortholog relationships inferred by Conant (2020) with the maximum log-likelihood value when the orthology/paralogy relationships between the zebrafish and stickleback paralog pairs are switched from those inferred by Conant (2020). Because it suggests problematic orthology/paralogy relationships, data sets were eliminated if the latter maximum likelihood value exceeded the former.

### The $\omega_{H}/\omega_{N}$ Model for Lineages that are not Postduplication

When analyzing the yeast and teleost data sets with the $\omega_{H}/\omega_{N}$ model, a choice has to be made about how to model the nonsynonymous rates prior to the duplication and also on the lineage to the outgroup. While other choices are possible, we chose to have $\omega_{N}$ be the nonsynonymous rate factor for these evolutionary intervals. Also, we normalized the rates for these evolutionary intervals to have one expected codon substitution arising from point mutation per time unit.

## Supplementary Material

msad198_Supplementary_Data

## Data Availability

Software and instructions for performing the IGC analyses are available at: https://github.com/xji3/IGCexpansion. Teleost and yeast data sets as well as output from IGC analyses are available at: https://github.com/Yixuan39/IGC-fish.
