# Supplementary material for: Interlocus Gene Conversion, Natural Selection, and Paralog Homogenization
Source: Mol Biol Evol. 2023 Sep 7;40(9):msad198. doi: 10.1093/molbev/msad198 (PMC10503786; doi:10.1093/molbev/msad198)
Supplement: msad198_Supplementary_Data [file msad198_supplementary_data.pdf]

Table 1: Supplemental Table: Teleost

Data Set Name	Length	$\widehat{\omega_N}$	$\widehat{\omega_H}$	$\widehat{\tau}$
Pillar211R	560	0.05	0.62	0.28
Pillar214R	581	0.06	0.37	0.18
Pillar222R	590	0.10	0.41	0.14
Pillar223R	379	0.09	0.44	0.14
Pillar337R	239	0.14	0.34	0.21
Pillar479R	775	0.11	0.41	0.09
Pillar521R	334	0.11	0.61	0.26
Pillar526R	304	0.16	0.45	0.10
Pillar735R	1490	0.04	0.41	0.23
Pillar1050R	320	0.08	0.28	0.08
Pillar1053R	408	0.07	0.59	0.09
Pillar2129R	500	0.15	0.39	0.17
Pillar2158R	837	0.06	0.32	0.30
Pillar2210R	395	0.10	0.82	0.22
Pillar2214R	641	0.12	0.39	0.11
Pillar2358R	285	0.09	0.60	0.05
Pillar2371R	296	0.12	0.61	0.06
Pillar2382R	526	0.07	0.38	0.12
Pillar2861R	204	0.03	0.23	0.18
Pillar3295R	229	0.07	0.44	0.18
Pillar3309R	134	0.09	0.69	0.34
Pillar3337R	566	0.12	0.31	0.19
Pillar3346R	786	0.10	0.50	0.19
Pillar3347R	466	0.05	0.70	0.28
Pillar3390R	253	0.13	0.50	0.13
Pillar3994R	281	0.13	1.34	0.69
Pillar4025R	1087	0.12	0.53	0.11
Pillar4031R	174	0.22	0.43	0.09
Pillar4063R	331	0.02	1.22	0.48
Pillar4268R	175	0.04	0.17	0.14
Pillar4494R	245	0.10	0.29	0.24
Pillar4553R	314	0.11	0.76	0.16
Pillar4570R	491	0.08	0.36	0.18
Pillar4932R	495	0.20	0.62	0.10
Pillar5233R	761	0.04	0.13	0.24
Pillar5316R	1886	0.09	0.48	0.12
Pillar5550R	255	0.11	0.60	0.13

Table 2: Supplemental Table: Yeast

Data Set Name	Length	$\widehat{\omega_N}$	$\widehat{\omega_H}$	$\widehat{\tau}$
YBL087C_YER117W_input	136	0.06	22026.47	2.26
YBR191W_YPL079W_input	159	0.08	3.17	3.37
YDR418W_YEL054C_input	163	0.08	18.59	0.97
YER074W_YIL069C_input	133	0.11	8.18	4.99
YER102W_YBL072C_input	198	0.17	7.53	3.01
YER131W_YGL189C_input	118	0.05	1.77	1.52
YJL177W_YKL180W_input	183	0.08	5.52	1.20
YLR333C_YGR027C_input	107	0.12	2.10	2.34
YLR406C_YDL075W_input	112	0.08	5.89	1.28
YML026C_YDR450W_input	140	0.08	22026.47	3.13
YMR142C_YDL082W_input	197	0.14	1.78	3.90
YMR143W_YDL083C_input	134	0.02	3.05	3.08
YNL069C_YIL133C_input	197	0.13	1.36	0.97
YNL301C_YOL120C_input	185	0.10	5.94	1.36
